# Impaired control of the oculomotor reflexes in Parkinson's disease

**DOI:** 10.1016/j.neuropsychologia.2009.06.018

**Published:** 2009-11

**Authors:** Martijn G. van Koningsbruggen, Tom Pender, Liana Machado, Robert D. Rafal

**Affiliations:** a Wolfson Center for Clinical and Cognitive Neuroscience, School of Psychology, Bangor University, Bangor, United Kingdom; b Department of Psychology and Brain Health and Repair Research Centre, University of Otago, Dunedin, New Zealand

**Keywords:** Eye movements, Anti saccade, Fixation offset effect, Cognitive control, Basal ganglia, Parkinson's disease, Oculomotor reflexes

## Abstract

To investigate the role of the basal ganglia in integrating voluntary and reflexive behaviour, the current study examined the ability of patients with Parkinson's disease to voluntarily control oculomotor reflexes. We measured the size of the fixation offset effect (the reduction in saccadic reaction time when a fixation point is removed) during a block of pro- and a block of anti-saccades. Healthy controls showed the expected reduction of the FOE during the anti-saccades, which results from efforts to suppress reflexive eye movements (a preparatory set characterized by increased internal control and reduced external control). However, there was no reduction of the FOE in the anti-saccade task in Parkinson's patients, indicating that they are impaired in exerting control over oculomotor reflexes.

The expansion of cerebral cortex in the course of evolution enabled a more flexible behavioural repertoire to adapt to more complex environments. This flexibility is achieved by adapting primitive reflexes as building blocks of more complex circuits, and orchestrating their employment in the service of goal directed behaviour (Easton, 1972; Rozin, 1976). Here we examined the role of the basal ganglia in implementing the facilitatory and inhibitory modulation of reflexes by studying the strategic control of oculomotor reflexes in patients with Parkinson's disease.

## 1 The anti-saccade task

A paradigm used frequently to study the ability of healthy individuals and different patient groups to control oculomotor reflexes is the anti-saccade task (Crawford, Bennett, Lekwuwa, Shaunak, & Deakin, 2002; Hutton & Ettinger, 2006; Munoz & Everling, 2004). In this paradigm, subjects are instructed to make a saccade in the opposite direction of a suddenly appearing peripheral visual stimulus, i.e. towards the mirror location. Correct performance depends on the combined ability to suppress an automatic saccade towards the sudden visual onset, and ability to initiate a voluntary saccade in the mirror direction (Hallett, 1978).

The suppression of saccades towards visual targets is achieved by exerting voluntary control over two primitive oculomotor reflexes: the visual grasp reflex (VGR), which moves the eyes towards a suddenly appearing target, and the fixation reflex, which anchors the eyes on a foveated stimulus. The superior colliculus (SC), which is considered to be the final common pathway for the different cortical and subcortical oculomotor areas (Moschovakis, Scudder, & Highstein, 1996), has been demonstrated to be involved in these two reflexes. There are two different types of neurons in the intermediate layers of the SC: fixation neurons, which give rise to the fixation reflex, and saccade neurons, which give rise to the VGR. The two reflexes mutually inhibit each other: more fixation related activity leads to less saccade activity, and vice versa (for a review see Munoz & Fecteau, 2002). Since there are no direct connections between these fixation and saccade neurons of the SC (Isa, 2002; Isa & Saito, 2001; Lee & Hall, 2006), the inhibitory interactions between these two reflexes could occur either downstream in the brainstem (Takahashi, Sugiuchi, Izawa, & Shinoda, 2005), or upstream in the oculomotor cortex (Dias & Bruce, 1994; Hanes, Patterson, & Schall, 1998) or substantia nigra *pars reticulata* (SNpr) (Basso & Liu, 2007; Hikosaka & Wurtz, 1983a, 1983b).

Neurophysiological studies have found that, compared to pro-saccades, during anti-saccades the activity of fixation neurons is enhanced, while the activity of saccade neurons is reduced in the SC and frontal eye field (FEF) (Everling, Dorris, Klein, & Munoz, 1999; Everling & Munoz, 2000). In addition, Everling, Dorris, & Munoz (1998) found that errors in the anti-saccade task, i.e. unsuppressed saccades towards the target, could be predicted by the amount of pre-target activity in saccade neurons within the SC, such that higher activity was correlated with more errors. These findings suggest that, by endogenously increasing the activity level of fixation cells and decreasing the activity level of saccade cells before the target appears, one is capable of suppressing the VGR.

## 2 The fixation offset paradigm

The fixation offset (FOE) paradigm (Kingstone & Klein, 1993; Saslow, 1967) can be employed to probe the amount of voluntary control over the fixation and visual grasp reflexes. The FOE refers to the reduction in saccadic reaction times when the fixation point disappears simultaneously with the target onset, compared to when the fixation point remains visible (overlap). This disappearance of the fixation point results in a decrease of activity of the fixation neurons (Munoz & Wurtz, 1992, 1993), which results in a relative disinhibition of the saccade neurons (Dorris & Munoz, 1995), i.e. less activity is needed to reach the saccade threshold. However, as noted earlier, the preparatory set that is adopted during a block of anti-saccades can bring these neurons under endogenous control.

Everling et al. (1999) showed that the strategic set requiring inhibition of the visual grasp reflex during anti-saccade task performance not only resulted in a tonic increase in fixation neuron activity, it also reduced stimulus-related activity in superficial layers of the superior colliculus: visual neurons in the rostal pole showed weaker stimulus-related responses during anti- compared to pro-saccade trials. Saccade neurons (buildup and burst) also showed weaker (and briefer) stimulus-related responses during anti- compared to pro-saccade trials. Thus, increased endogenous control during anti-saccades renders collicular neurons less responsive to external visual signals both in the periphery (attenuating the VGR) and at fixation, thereby attenuating the fixation reflex, and reducing the FOE (Forbes & Klein, 1996; Machado & Rafal, 2000a; Reuter-Lorenz, Hughes, & Fendrich, 1991).

The difference between the size of the FOE during pro-saccades and anti-saccades can therefore be used to measure the amount of control over the fixation and visual grasp reflexes: more voluntary control leads to a greater decrement in the FOE for anti-saccades compared to the FOE for prosaccades. In the current investigation we compared the magnitude of the FOE in prosaccade and anti-saccade tasks to examine the ability of patients with Parkinson's disease to exercise strategic control over oculomotor reflexes.

## 3 Oculomotor control and the basal ganglia

There is evidence that there are at least 9 different loops from the basal ganglia, which are dysfunctional in PD patients, to different cortical areas, including primary motor, pre-motor, FEF, prefrontal, and inferotemporal cortex. One such a loop is referred to as the oculomotor loop (Alexander, DeLong, & Strick, 1986; Galvan & Wichmann, 2008; Middleton & Strick, 2000a, 2000b). The input area of the oculomotor loop is the caudate nucleus, which receives input from different areas of the oculomotor cortex: FEF, dlPFC, supplementary eye fields and parietal cortex. The caudate nucleus is connected to the SNpr via direct inhibitory projections, and indirect net excitatory projections. The SNpr has direct inhibitory projections to the intermediate layers of the SC (Hikosaka & Wurtz, 1983b). Hikosaka and Wurtz (1983a) showed that neurons in the SNpr responded to stimuli in their receptive field with a decrease in spike frequency. Hikosaka and Wurtz (1983a) also examined the effect of a fixation target, which was presented centrally and not in their receptive field. They found that the neuronal response was increased when the monkeys were fixating, indicating that this pathway is involved in fixation related processes. Further evidence for this involvement has been provided by a recent study that used electrical stimulation to disrupt SNpr cells (Basso & Liu, 2007). Short bursts of electrical stimulation decreased the latency of visually guided saccades, whereas the latencies of memory guided saccades increased. In addition, the SNpr not only projects to the SC, it is also connected to thalamic nuclei, which project back to the FEF. SNpr can therefore inhibit the activity of saccade neurons of the SC and the FEF (Alexander et al., 1986; Middleton & Strick, 2000b; Munoz & Everling, 2004).

The role of the basal ganglia in controlling oculomotor reflexes has been inferred from observations of the development of human infants. The fixation reflex, which inhibits the VGR, is extremely powerful in babies. After around 2 months the SNpr starts to exert inhibitory control over the SC, leading to ‘sticky fixations’, i.e. infants exhibiting difficulty moving their eyes (Atkinson, Hood, Wattam-Bell, & Braddick, 1992; Hood, Atkinson, Braddick, & Wattam-Bell, 1992; Johnson, 1990). McConnell and Bryson (2005) tested 25 infants at 2, 4, and 6 months of age. The results demonstrated that the latencies for overlap trials showed a massive decline between 2 and 4 months. Furthermore it has been suggested that voluntary control over the VGR and fixation reflex improves up to 20 years of age (Fischer, Biscaldi, & Gezeck, 1997). In other words, the maturation of fronto-nigral-collicular pathways results in voluntary control over this reflex. Damage to these circuits may lead to a reduction in, or even loss of, this voluntary control. In the case of Parkinson's disease, the loss of dopamine in the striatum might compromise the ability to control oculomotor reflexes.

A recent study investigated the size of FOE for both reflexive and voluntary saccades in patients with a lesion to the pulvinar, and PD patients (Rafal, McGrath, Machado, & Hindle, 2004). Reflexive saccades were made to peripheral targets and voluntary saccades were made in response to verbal instructions (i.e. ‘left’ or ‘right’). Fixation offset and overlap trials were randomized within blocks. Pulvinar lesioned patients had a reliable FOE during voluntary saccades that was comparable to healthy controls, but not during reflexive saccades. This implies that different neural systems control fixation when making voluntary and visually triggered eye movements. However, more relevant to the current study, saccade latencies and FOE magnitudes of PD patients were comparable to healthy controls for both types of saccade, indicating that PD patients do not differ from healthy controls when the strategic set does not entail inhibiting eye movements.

Chan, Armstrong, Pari, Riopelle, and Munoz (2005) tested 18 PD patients on separate blocks of pro-saccades and anti-saccades. A gap manipulation, in which the fixation point disappears 200 ms before the target onset or remains visible (overlap), was included in both blocks (Saslow, 1967). It has been proposed that the gap effect, which is larger than the FOE, is a combination of the effect of a general warning signal and a FOE (Forbes & Klein, 1996; Kingstone & Klein, 1993). PD patients made more express saccades in the pro-saccade task during both the overlap and gap trials compared to healthy controls. PD patients also made more directional errors in the anti-saccade task, and were slower to initiate a saccade in the opposite direction. Their results suggest that PD patients have more difficulties suppressing automatic responses. Although the authors did not report whether the size of the gap-effect was influenced by the strategic set (i.e. pro- versus anti-saccades), comparison of their Tables 1 and 2 indicate that neither PD patients nor healthy controls showed a reduction in the gap effect for anti- versus pro-saccades. However, given that the gap effect reflects both the FOE and a general warning effect, it is unclear whether modulation of the FOE changes with PD.

Amador, Hood, Schiess, Izor, and Sereno (2006) tested PD patients on anti-saccades, delayed anti-saccades, and remembered anti-saccades. Compared to controls, the patients were slower to initiate saccades on all tasks, and had more difficulties inhibiting automatic responses. These results were predicted on the basis of their theoretical model, the tonic inhibition model of orienting (Sereno, 1992). The tonic inhibition model posits that there is a voluntary and reflexive attentional system. The voluntary system, which consists of the prefrontal cortex and basal ganglia, has a tonic inhibition over the reflexive system (brainstem and colliculi). The model further predicts that an impaired voluntary system would lead to a disinhibited reflexive system. In a recent study by the same group, the effect of the dopamine pre-cursor levodopa, an often prescribed medication for PD patients, was examined on saccade performance (Hood et al., 2007). Patients were tested on two occasions. During the first session, patients were tested at least 12 h after their last levodopa medication, i.e. in the off state. During the second session, patients were tested while medicated, i.e. in the on state. They were tested on a block of pro- and a block of anti-saccades, both with a gap manipulation. Interestingly, levodopa medication resulted in longer latency pro-saccades, and less errors during the anti-saccade task. However, the authors did not report any statistical tests considering the size of the gap effect. Neither PD patients nor controls appear to show a reduction in the *gap effect* for anti- compared to pro-saccades. Once again, given that the gap effect reflects both the FOE and a general warning effect, it is not possible to determine from this study whether strategic control of ocular fixation is compromised in patients with PD.

To summarize, compared to controls, PD patients appear to make more direction errors on the anti-saccade task (Amador et al., 2006; Armstrong, Chan, Riopelle, & Munoz, 2002; Briand, Strallow, Hening, Poizner, & Sereno, 1999; Chan et al., 2005; Crevits & De Ridder, 1997; Hood et al., 2007; Kitagawa, Fukushima, & Tashiro, 1994) which could be caused by a general impairment of saccade suppression (Chan et al., 2005). In addition some studies have reported longer saccade latencies of anti-saccades in PD patients. However, no studies have directly studied whether PD patients can endogenously control the size of the FOE. The specific goal of the current study was to investigate the ability of PD patients in controlling oculomotor reflexes by comparing the magnitude of the FOE for prosaccades and anti-saccades.

## 4 Methods

### 4.1 Participants

Nineteen non-demented (all MMSE > 27) patients with PD (mean age = 66.56; SD = 6.71) and twenty age matched controls (mean age = 66.10; SD = 5.09) were tested. The patients were diagnosed with PD on average 7.06 years (SD = 4.95) prior to testing. The Unified Parkinson's Rating Scale was administered to all patients (mean score = 15.28; SD = 7.81). All patients were tested while on medication. None of the PD patients had implants; see Table 1 for more clinical details. The study was approved by the ethics committees of the School of Psychology, Bangor University, and the North-West Wales NHS.

### 4.2 Stimuli and procedure

Presentation software (Neurobehavioral Systems) was used to present the stimuli on a Mitsibuthsi Super Bright CRT Monitor (240 Hz), which was 57 cm in front of the subjects. Horizontal eye position was recorded with an Eye Trac 210 scleral reflectance device (ASL) at a sampling rate of 1000 Hz. The analogue output of the right eye was recorded by a Powerlab data acquisition unit (ADInstruments) and stored for off-line analyses.

Throughout the experiment, two white marker boxes (1.5°) on a black background were presented at 9° to the left and right of the centre of the screen. After an inter-trial interval of 2500 ms, each trial began with the onset of a fixation point, a 0.4° white filled circle, in the centre of the screen. After the fixation point onset, the experimenter, who was present throughout the whole experiment, started the trial only when the participant was looking at the central fixation point. If the participant was not looking at the fixation point, the experimenter would ask the participant to look at the fixation point. A 1000 Hz sound (100 ms) was presented as soon as the experimenter initiated the trial, and served as a general warning signal for the participants. After a randomized delay between 250 and 750 ms (in steps of 25 ms), the left (50%) or right (50%) marker box turned white. On half of the trials, the fixation point remained visible (overlap condition), while on the other half it disappeared simultaneously with the onset of the visual target (offset condition). The target remained on the screen for 750 ms. Participants were instructed to make an eye movement to the centre of this box as fast as possible during the pro-saccade task, and instructed to make an eye movement towards the centre of the opposite box during anti-saccades. The experimenter constantly monitored the performance, and provided feedback to the participant on every trial.

Every session started with 10 practice trials. The main experiment was only started if the participant understood the task, and made less than 50% errors, otherwise additional practice trials were presented. A total of 100 test trials were presented for each task, with a three point calibration every 10 trials and after every significant head movement. Each task took approximately 45 min to complete. The patients completed the pro-saccades and anti-saccades on different days due to the length of the experiment. In addition regular breaks were interspersed to ensure good task performance. The healthy controls completed both task during one session, with anti-saccades and prosaccades in different blocks. The task was explained to the subject at the start of each block. The task order was counterbalanced across subjects.

### 4.3 Data analyses

Matlab was used to analyze the eye movement data. First the horizontal position signal was filtered with a 3-ms FWHM (full width at half maximum) Gaussian kernel filter to remove noise. Next, the velocity profile was calculated. The first sample with a velocity greater than 30 degrees per second, if followed by an increasing velocity over the next 10 samples, was marked as the saccade onset. The saccade offset was determined based on similar criterion: the first sample with a velocity smaller than 30 degrees per second, and a decreasing velocity profile in the preceding 10 samples. All eye movement traces were visually inspected by the experimenter to determine whether the algorithm had identified the onset and offset correctly, and whether the eye movements were not contaminated by blinks. Trials were rejected by the experimenter from further analyses if the algorithm was incorrect, or the eye movement was contaminated by blinks. In addition, eye movements with a reaction time shorter than 75 ms or longer than 750 ms, that did not start within ±1 degree of central fixation, and with amplitudes of less than 6 degrees or more than 14 degrees were also rejected. Based on these criteria, significantly more trials were rejected for PD patients (16%) than for healthy controls (8%), *F* (1,37) = 11.23, *p* < 0.01, $\eta_{\text{p}}^{2}=0.23$. However, the amount of rejected trials did not differ for pro- and anti-saccades (*p* = 0.17), nor was there a significant interaction between the task and the group (*F* < 1).

### 4.4 Reaction time analyses

**Fig. 1:**
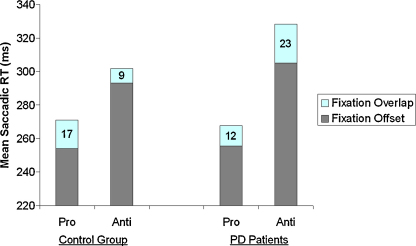
Mean Saccadic Reaction times for both groups, with the size of the FOE.

Trials on which a direction error was made were excluded from the saccadic RT analyses. Since a preliminary analyses showed no difference in RT for left and right eye movements (*p* > 0.2) data for left and right eye movements were pooled. The mean saccade latencies and the size of the FOE are displayed in Fig. 1. Kolmorgorov-Smirnov tests indicated that most variables deviated from normal distribution, which was resolved by a LOG-transformation. Therefore, all statistical tests are based on the LOG-transformed data. However, graphs and reported reaction times are based on the mean reaction times. Means of the log saccade latency for correct responses were calculated in each condition for each participant and subjected to a repeated measures analyses of variance (ANOVA) with the task (Pro-saccades vs. Anti-saccades), and Fixation point condition (offset and overlap) as within subject factors, and Group (PD patients vs. Healthy controls) as between subject factors. There was no significant difference between the two Groups, *F* (1,37) < 1. The main effect for Task was significant, *F* (1,37) = 57.78, *p* < 0.001, $\eta_{\text{p}}^{2}=0.61$, indicating that reaction times for anti-saccades (307 ms) were longer than for pro-saccades (262 ms). In addition, the main effect of Fixation Point Condition was significant, *F* (1,37) = 30.93, *p* < 0.001, $\eta_{\text{p}}^{2}=0.46$, caused by shorter saccadic latencies for fixation point offset trials (277 ms) compared to overlap trials (292 ms). The interactions between Task × Fixation Point Condition, and between Task × Group were not significant (both *F* < 1). More important, the three-way interaction between Task × Fixation Point Condition × Group was significant, *F* (1,37) = 6.03, *p* < 0.05, $\eta_{\text{p}}^{2}=0.14$.

Two paired samples *t*-tests were conducted to further investigate the three-way interaction. The size of the FOE during anti-saccades (FOE = 9 ms) was significantly smaller than during pro-saccades (FOE = 17 ms) for the control group, *t* (19) = 2.41, *p* = 0.01. However, for PD patients, the size of the FOE did not depend on whether participants were performing anti-saccades (FOE = 23 ms) or pro-saccades (FOE = 12 ms), *t* (18) = −1.17, *p* = 0.87.

**Fig. 2:**
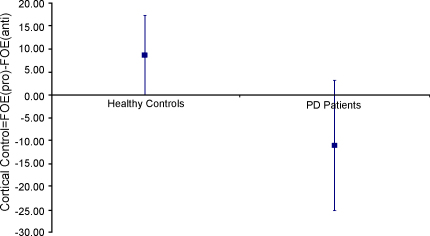
The 95%CI of the mean amount of control over oculomotor reflexes (=FOE_(Pro-Saccades)_ − FOE_(Anti-Saccades)_) for both PD patients and healthy controls.

The amount of cortical control can be estimated by calculating the difference between the size of the FOE during pro- and anti-saccades: Control = FOE_(Pro-Saccades)_ − FOE_(Anti-Saccades)_. A larger value reflects more control. The 95% confidence interval for the amount of control for both PD patients and healthy controls is shown in Fig. 2. A *t*-test confirmed that healthy controls had more control (Control = 9 ms) than PD patients (Control = −11 ms), *t* (37) = 2.5, *p* < 0.05.

The interaction between Task and Group was not significant, which was not expected since it has been frequently reported that PD patients are slower to initiate anti-saccades compared to healthy controls. To further investigate whether PD patients were slower during the anti-saccade task, two independent samples *t*-tests were conducted to compare the saccade latencies for both overlap and offset trials between PD patients and healthy controls. However, there were no significant differences between PD patients and controls for either the anti-saccade overlap trials (*p* = 0.15) or the anti-saccade offset trials (*p* = 0.27).

### 4.5 Saccade amplitude analyses

**Fig. 3:**
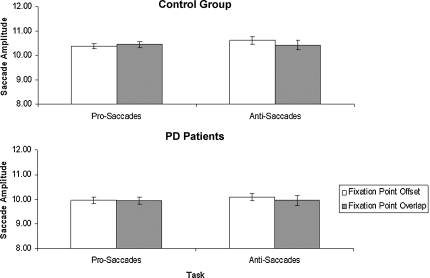
Mean saccade amplitude for each condition (±1SEM) for both the healthy controls (top panel) and PD patients (bottom panel).

Correct eye movements were also analyzed for amplitude. Since a preliminary analysis showed no difference in amplitude for left and right eye movements (*p* > 0.4), data for left and right eye movements were pooled. Mean saccade amplitude was calculated in each condition for each participant and subjected to a repeated measures ANOVA with the task (Pro-saccades vs. Anti-saccades), and Fixation point condition (offset and overlap) as within subject factors, and Group (PD patients vs. Healthy controls) as between subject factors. The saccadic amplitude was significantly smaller for PD patients (9.81 degree) than for healthy controls (10.46 degree), *F* (1,37) = 6.78, *p* = 0.01, $\eta_{\text{p}}^{2}=0.16$. The main effect of task was not significant, *F* (1,37) = 1.05, *p* = 0.31, $\eta_{\text{p}}^{2}=0.03$, indicating that there was no difference between the amplitude of pro- and anti-saccades. There was no amplitude difference between offset and overlap trials, *F* (1,37) = 1.23, *p* = 0.27, $\eta_{\text{p}}^{2}=0.03$. There were no significant interactions between Task × Group, Fixation Point Condition × Group, and Task × Group × Fixation Point Condition (all *F*'s < 1). However, the interaction between Task and Fixation Point Condition was significant, *F* (1,37) = 4.53, *p* = 0.04, $\eta_{\text{p}}^{2}=0.11$. Paired wise comparisons revealed no significant differences. The mean saccade amplitudes are shown in Fig. 3.

### 4.6 Direction error analyses

As expected, most subjects did not make any direction errors during the pro-saccade task. Since, on average, both PD patients and healthy controls never made more than 1% direction errors during the pro-saccade task, this condition was not further analyzed.

**Fig. 4:**
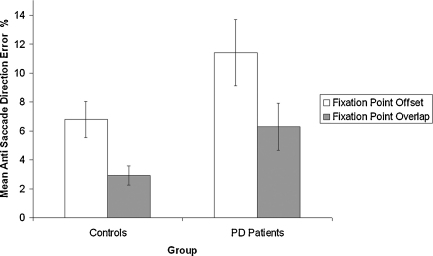
Mean amount of direction errors during the anti-saccade task (±1SEM).

Therefore, only the direction errors during anti-saccades were analyzed. Data for both right and left eye movements were pooled, since a preliminary analyses showed no difference (*p* > 0.5). Mean direction errors were calculated in each condition for each participant and subjected to a repeated measures ANOVA with Fixation point condition (offset and overlap) as within subject factors, and Group (PD patients vs. Healthy controls) as between subject factors. PD patients made significantly more direction errors (8.8%) than healthy controls (4.8%), *F* (1,37) = 4.29, *p* < 0.05, $\eta_{\text{p}}^{2}=0.10$. The main effect of Fixation Point condition was also significant, *F* (1,37) = 18.87, *p* < 0.01, $\eta_{\text{p}}^{2}=0.34$. This was caused by the fact that subjects made more errors during the fixation point offset condition (9.1%) compared to the fixation point overlap condition (4.6%). However, the two-way interaction between fixation point condition × group was not significant (*F* < 1). The mean anti-saccade direction errors are shown in Fig. 4.

## 5 Discussion

The current study investigated whether PD patients can exert normal control over their oculomotor reflexes. The size of the FOE, i.e. the difference in saccadic RT between overlap and offset trials, was measured during both a pro-saccade task and an anti-saccade task. Healthy controls were able to endogenously control oculomotor reflexes, as reflected by a decrease in the size of FOE during anti-saccades compared to pro-saccades. However, this form of cognitive control was absent in the PD patients. Impaired control was manifest both as greater errors (compared to controls) and the failure to reduce the FOE: the size of the FOE did not depend on the type of eye movement. This indicates that PD patients cannot employ the same preparatory set in regulating the responsiveness of fixation neurons to visual signals.

Since successful anti-saccade performance requires both suppression of a reflexive saccade, and the generation of a voluntary saccade, one or both of these steps could be affected in PD patients. However, given that a previous study found that PD patients have a normal FOE for both reflexive and voluntary pro-saccades (Rafal et al., 2004), it seems that the preparatory set required to suppress the VGR is non-normal in PD patients.

PD patients were not significantly slower during anti-saccades than healthy controls. However, consistent with previous research (Briand et al., 1999; Crawford et al., 2002), PD patients made significantly more direction errors during anti-saccades, suggesting that impaired control resulted in a speed-accuracy trade off. Other explanations for the failure to find differences in anti-saccade latencies between PD patients and healthy controls include the fact that our patients were tested while on medication, or the fact that a warning signal was presented at the start of the trial.

A recent review considers the contradictory evidence regarding PD performance on the anti-saccade task (Crawford et al., 2002). They hypothesize that these discrepancies could be caused by the fact that PD patients form a heterogeneous group. It has been reported that some PD patients show similar impairments on cognitive tasks as patients with a lesion to the frontal lobe (Dubois & Pillon, 1997), which could be caused by a depletion of dopamine in the prefrontal cortex (Crawford et al., 2002; Scatton, Javoy-Agid, Rouquier, Dubois, & Agid, 1983). To test their hypothesis, Crawford, Haeger, Kennard, Reveley, and Henderson (1995a, 1995b) tested PD patient on an anti-saccades task, and tested their frontal lobe function on the Wisconsin Card Sort Test. They discovered that anti-saccade performance was highly correlated with preservative errors on the Wisconsin Card Sort Test.

The finding of the current study, that PD patients cannot endogenously control the FOE, indicates that the basal ganglia are involved in exercising this control. As discussed in the introduction, the basal ganglia participate in different cortical loops, one of which is referred to as the oculomotor loop (Alexander et al., 1986; Galvan & Wichmann, 2008; Middleton & Strick, 2000a). Patients with a lesion to the FEF are also impaired in controlling the same kind of oculomotor reflexes (Machado & Rafal, 2004a, 2004b), suggesting that the FEF is needed for this control. Additional evidence for the involvement of the FEF is provided by Connolly, Goodale, Menon, and Munoz (2002). They studied preparatory set in the human oculomotor cortex using fMRI. They measured the BOLD activity in both the FEF and intraparietal sulcus during a response preparation period (i.e. no actual response was generated). They found that the FEF shows greater preparatory activity for anti-saccades than for pro-saccades. In an additional study, they showed that the pre-target FEF activation correlated with subsequent anti-saccade RT (Connolly, Goodale, Goltz, & Munoz, 2005). Further evidence is provided by a TMS study. Olk, Chang, Kingstone, and Ro (2006) tested subjects on a modified anti-saccade task, in which pro- and anti-saccades are mixed within a block, and inhibition was required for both pro- and anti-saccades. TMS over the FEF increased the latencies of anti-saccades directed ipsilaterally but did not influence the latencies of pro-saccades. The FEF has direct connections to the SC, and indirect connections to the SC via the SNpr of the basal ganglia (Moschovakis et al., 1996). The reduced control over the FOE could be the result of the disrupted basal ganglia route. However, the basal ganglia also project back to the FEF, which could result in a relatively dysfunctional FEF.

Recent evidence has suggested that the monkey DLPFC is also involved in anti-saccade tasks. Johnston and Everling (2006) measured from a subset of neurons in the monkey DLPFC, that had direct connections with the SC. Like FEF and SC neurons, the DLPFC neurons showed higher pre-target activity during anti- than during pro-saccades, and presaccadic activity that correlated with anti-saccade reaction times. There is also evidence that the human DLPFC is involved in the anti-saccade task. Nyffeler et al. (2007) reported that TMS over the DLPFC 100 ms before the target resulted in more erroneous reflexive saccades towards the target. In addition, patients with lesions involving the DLPFC have an increased error rate on the anti-saccade task (Pierrot-Deseilligny, Muri, Nyffeler, & Milea, 2005). Dysfunction of the DLPFC might also be implicated in impaired control of oculomotor reflexes, either due to dopamine deficiency in the part of the basal ganglia receiving projections from it, or due to dopamine deficiency within the DLPFC itself (Scatton et al., 1983).

Endogenous control over oculomotor reflexes results in a reduced FOE because it renders the activity of fixation neurons less contingent upon the presence of a stimulus at fixation. In the anti-saccade task, control is exerted as inhibition. However an increase in readiness to make saccades can also reduce the FOE. In the prosaccade task, for example, the FOE can be reduced by decreasing the proportion of catch trials (i.e. where no saccade target appears) (Machado & Rafal, 2000b), or by providing a precue instructing subjects to prepare a saccade to a specified location (Machado & Rafal, 2004b; Rafal, Machado, Tony, & Ingle, 2000). In these cases the reason for the reduced FOE is transparent: oculomotor readiness induces subjects to reduce fixation neuron activity *before* the target appears, so the presence or absence of the fixation point has less influence.

The reason for the reduced FOE in the anti-saccade task is less obvious. A tonic increase in fixation cell activity cannot, by itself, explain this effect (since this is true for both offset and overlap trials). A reduced FOE requires that the strategic set also reduce the responsiveness of fixation neurons to visual stimuli in their receptive field. As noted in the introduction, collicular neurons are less responsive to visual stimuli during the anti-saccade task. At this point, however, there is no direct evidence that this reduced responsiveness to visual stimuli is responsible for the reduction in the FOE. In a gap paradigm, Bell, Everling, and Munoz (2000) reported that fixation cell activity decreased, in the offset condition, to the same degree in prosaccade and anti-saccade tasks. However, the gap effect also did not differ for prosaccades and anti-saccades, perhaps because preparation from a warning signal masks the attenuating effect of anti-saccade preparation on the FOE (Dick, Kathmann, Ostendorf, and Ploner, 2005).

In conclusion, the current comparison of the FOE in prosaccades and anti-saccades implicates the basal ganglia, or dopaminergic influences on cortex, in the control of oculomotor reflexes. The physiological basis for the reduction of the FOE during anti-saccades requires further study at the level of collicular neurons using an FOE paradigm; and the circuitry disrupted in Parkinson's disease that leads to the loss of control remain to be specified.

## Figures and Tables

**Table 1:** 

Patient	Sex	Age	Disease duration	UPDRS	Medication
1	F	69	6	27/92	Prarnipexole
2	M	64	13	24/92	Levodopa-Carbidopa
3	M	64	12	7.5/92	Ropinerole, Levodopa-Carbidopa
4	M	59	2	6.5/92	Ropinerole, Rasagiline
5	F	70	5	19.5/92	Levodopa-Carbidopa, Prarnipexole
6	M	67	5	15/92	Prarnipexole
7	M	77	12	15192	Levodopa-Carbidopa, Prarnipexole
8	M	62	7	U.5/92	Levodopa-Carbidopa, Prarnipexole
9	M	61	17	19.5/92	Prarnipexole, Benserazide, Entacapon
10	M	55	44	3/92	Prarnipexole
11	M	61	5	16/92	Levodopa-Carbidopa
12	F	*59*	18	17/92	Ropinerole,
13	M	63	3	10/52	Ropinerole
14	F	71	3	9.5/92	Levodopa-Carbidopa
15	F	71	6	16.5/92	Levodopa-Carbidopa, Ropinerole
16	M	81	11	21/92	Levodopa-Carbidopa
17	F	68	2	30/92	Levodopa-Carbidopa
18	M	72	6	3/92	Levodopa-Carbidopa, Prarnipexole
